# Supplementary material for: A terahertz meta-sensor array for 2D strain mapping
Source: Nat Commun. 2024 Apr 11;15:3157. doi: 10.1038/s41467-024-47474-3 (PMC11009334; doi:10.1038/s41467-024-47474-3)
Supplement: Supplementary file 1 — Supplementary Information [file 41467_2024_47474_MOESM1_ESM.pdf]

A terahertz meta-sensor array for 2D strain mapping

Table of contents

■ Supplementary Figure List

Fig. S1. Simulation results of ED and MD resonances versus microsphere spacing (P_x and P_y) in terms of ideal vacuum conditions.

Fig. S2. Illustration of near-field coupling among four neighboring ZrO_2-microsphere resonators.

Fig. S3. Measured transmission spectra of a pure PDMS with the identical thickness to our device under different external strains.

Fig. S4. Effect of the PDMS on ED and MD resonances.

Fig. S5. Fabrication process of the flexible strain meta-sensor array.

Fig. S6. Simulated transmission spectra of meta-sensor arrays at 70% and 75% strain, and E-field distributions (@ the corresponding ED resonances) across the centre *x*-*y* plane of the microspheres as ε_y = 70% (i) and ε_y = 75% (ii).

Fig. S7. Cyclic tensile testing of the fabricated meta-sensor.

Fig. S8. Statistical analysis for the minimum strain variations that can be detected by our device.

Fig. S9. Simulated transmittance spectra of the meta-sensor array simultaneously applied with bidirectional tensile strain.

Fig. S10. Measured THz transmission spectra of the sample stretched along different angles.

Fig. S11. Demonstration of the bidirectional tensile test.

Fig. S12. Characterization of the meta-sensor array samples with self-cleaning capacity.

Fig. S13. Unidirectional tensile strain detection performance evaluation of the reflective meta-sensor.

Fig. S14. Bidirectional tensile strain detection performance evaluation of the reflective meta-sensor.

Fig. S15. Simulated reflection spectra of the reflective meta-sensor array simultaneously applied with *x*- and *y*-directional tensile strain.

Fig. S16. Measured resonance-strain response at the different locations of the fabricated sample.

Fig. S17. Demonstration of the unidirectional tensile test.

Fig. S18. Bending response test for the meta-sensor array.

Fig. S19. Twisting response test for the meta-sensor array.

■ Supplementary Table List

Table S1. Comparison of our meta-sensor array with previous representative strain sensor arrays works mentioned in Fig. 1c of the main text.

Table S2. Measured MD and ED resonance information across the 10 × 10 girds on the sample surface by 2D strain mapping.

Table S3. Calculation of the strain values across the 10 × 10 girds on the sample surface by means of visual computational analysis (original length and the length after strain of the mesh for *y* direction).

Table S4. Calculation of the strain values across the 10 × 10 girds on the sample surface by means of visual computational analysis (original length and the length after strain of the mesh for *x* direction).

■ Supplementary Note List

Note S1. Mie resonance theory.

Note S2. Analysis of physical mechanisms of strain-dependent THz response based on electromagnetic coupling between microspheres.

Note S3. The effect of PDMS on ED and MD resonances.

Note S4. Fabrication of the flexible strain meta-sensor array with self-cleaning capability.

Note S5. Analysis of the minimum detectable strain variations of our device.

Note S6. Calculation of the *GF* of our device.

Note S7. Self-cleaning effect of the meta-sensor array.

Note S8. Design and performance evaluation of a reflection-type meta-sensor for external strain detection.

Note S9. Bending deformation response of the meta-sensor array.

Note S10. Twisting deformation response of the meta-sensor array.

■ Supplementary References

Table S1. Comparison of our meta-sensor array with previous representative strain sensor arrays works mentioned in Fig. 1c of the main text.

Device type	Sensitive material	Electrode and interconnects	Stretchability	Element Size	Array scale	Orientation recognition	Mapping strain	References
Resistive	AgNW	Cu	30%	~25mm	3 × 3	-		S1
	AgNW/PDMS	Ag paste, CB-PDMS/Ecoflex	10%	Conductive electrode (3 × 3.5 mm^2); sensitive film (6 × 2 mm^2)	4 × 9	-		S2
	AgNW-PDMS	Liquid metal	35%	Each pixel was composed of a 5× 5 mm^2 metal layer island with 2.5 mm intervals	4 × 4	√		S3
	Ecoflex	electrical lines	5%	~5 × 5 cm^2	3 × 3	-		S4
	MWCNT/EA resin	MWCNT/EA-2	60%	~1 × 1 cm^2	4 × 4	-		S5
	Conductive hydrogels	Copper tapes	200%	~5 × 5 mm^2	6 × 8	-		S6
	Thin film of ZnO microparticles	Copper tape	0.8%	Electrode size ~5 × 5 mm^2, pitch size 9 mm	4 × 4	-		S7
	Constantan	Ag thin films	300%	5 × 5 mm^2	10 × 10	-		S8
Resistive +Field effect transistors (FOTs)	Porous graphite structures	Stretchable transistor; Carbon-nanotube electrodes	100%	2 × 2 mm^2	10 × 10	-		S9
	self-healable semiconducting film	Au and polystyrene-block-poly(ethylene-ran-butylene)-block-polystyrene (SEBS) elastomer	100%	~3 × 3 mm^2	5 × 5			S10
Resistive +strain gauge	Au/PU-PDMS nanomeshes	AgNWs	60%	~3 × 8 mm^2.	18	-		S11
Magnetic material	Ferromagnetic cobalt layer and ferromagnetic permalloy (NiFe) layer	Cu and NiFe layers	120%	Micrometre-sized area	-	√		S12
Capacitive	Ecoflex	Ag, Copper wire	50%	~1 × 1 cm^2	3 × 3	-		S13
	Ecoflex	CB-Ecoflex	500%	~30 × 130 mm^2	2 × 3	-		S14
	Ion conductor	Stretchable electrode; Ag nanowires	50%	~5 × 5 mm^2	10 × 10	-		S15
piezoelectric effect	Poly(vinylidene fluoride-co-trifluoroethylene) (P(VDF-TrFE))	Graphene , Au and ion gel	0.3%	~6 × 6 mm^2	4 × 4	-		S16
	PZT piezoelectric elements	Cr/Au	0.035%	~5 × 5 mm^2	3 × 3	-		S17
Plasmonic resonance	PDMS; Au layer	Non-contact type	~100%	-	-	Orthogonal direction	-	S18
	PDMS; Au layer	Non-contact type	< 10%	-	-	Orthogonal direction	-	S19
	PDMS; Au layer	Non-contact type	~2%	-	-	-	√	S20
**Mie resonance**	**ZrO_2 array**	**-**	**156%**	**~3×3 mm^2**	**~36 × 43**	**Arbitrary direction (15°, 30°, 45°, 60°, 75°)**	√	**This work**

Note S1. Mie resonance theory.

The generation of resonances can be explained and predicted by Mie theory[S21–S23]. A nonmagnetic dielectric sphere of radius r and refractive index n, ε_p and μ_p are its permittivity and permeability, respectively, where $\varepsilon_p = n^2$. The permittivity and permeability of the background material on which the dielectric sphere is located are denoted by ε_h and μ_p. Considering the first level electric and magnetic dipole resonance, the scattering coefficients of electric and magnetic dipoles can be expressed by a_1, b_1 respectively as

$$a_1 = \frac{n\psi_1(nx)\psi'_1(x) - \psi_1(x)\psi'_1(nx)}{n\psi_1(nx)\xi'_1(x) - \xi_1(x)\psi'_1(nx)} \tag{1}$$

$$b_1 = \frac{\psi_1(nx)\psi'_1(x) - n\psi_1(x)\psi'_1(nx)}{\psi_1(nx)\xi'_1(x) - n\xi_1(x)\psi'_1(nx)} \tag{2}$$

where $\psi(x)$, and $\xi(x)$ are the Riccati-Bessel functions.

From the Clausius-Mossotti formula, it follows that, in the case of spherical dielectric particles with dimensions much smaller than the wavelength of the incident electromagnetic wave their equivalent permittivity and equivalent permeability can be expressed as

$$\varepsilon_{eff} = \varepsilon_h \frac{2(k_0 r_0)^3 + 6ia_1 f}{2(k_0 r_0)^3 - 3ia_1 f} \tag{3}$$

$$\mu_{eff} = \varepsilon_h \frac{2(k_0 r_0)^3 + 6ia_1 f}{2(k_0 r_0)^3 - 3ia_1 f} \tag{4}$$

where $f = 4\pi N r_0^3 / 3$ is the volume fraction of the spherical media particles and N is the unit density of the microsphere particles. Also based on the Mie medium scattering theory, the above equations are combined to give

$$a_1 = m\frac{2}{3}(k_0^2 \varepsilon_h \mu_h)^{\frac{3}{2}} \frac{\varepsilon_h - \varepsilon_r F(\theta)}{2\varepsilon_h + \varepsilon_r F(\theta)} r^3 \tag{5}$$

$$b_1 = m\frac{2}{3}(k_0^2 \varepsilon_h \mu_h)^{\frac{3}{2}} \frac{\mu_h - \mu_r F(\theta)}{2\mu_h + \mu_r F(\theta)} r^3 \tag{6}$$

$$F(\theta) = \frac{2(\sin\theta - \theta\cos\theta)}{(\theta^2 - 1)\sin\theta + \theta\cos\theta} \tag{7}$$

$$\theta = k_0 r\sqrt{\varepsilon_r \mu_r} \tag{8}$$

(1) Electric dipole resonance

When a_1 is infinite,the subwavelength spherical particle can excite a strong electric dipole, then from the Eq. (5) can be

$$F(nx) = -2\frac{\varepsilon_h}{\varepsilon_p} \quad (9)$$

If $|\varepsilon_p| \gg |\varepsilon_h|$, that is to say, if the dielectric constant of the dielectric sphere is much larger than the dielectric constant of the surrounding medium, it can be obtained by Eq. (9):

$$F(nx) \approx 0 \quad (10)$$

Substituting above Equation into Eq. (7) the equation, we get:

$$\tan(nx) = nx \quad (11)$$

The first correct solution of the above formula is n=4.49, from which the minimum resonance frequency of the electric dipole can be calculated as follows

$$f_E = \frac{4.49c}{2\pi nr} \quad (12)$$

(2) Magnetic dipole resonance

When b_1 is infinite, the subwavelength spherical particles can excite strong magnetic dipoles, then it can be obtained by Eq. (6)

$$F(nx) = -2 \quad (13)$$

Taking above equation into Eq. (7) gives

$$\sin(nx) = 0, nx \neq 0 \quad (14)$$

The root of Eq. (14) is:

$$nx = \pi q, q = 1,2,3,... \quad (15)$$

When q=1, the frequency of the minimum resonance of the magnetic dipole is

$$f_H = \frac{c}{2nr} \quad (16)$$

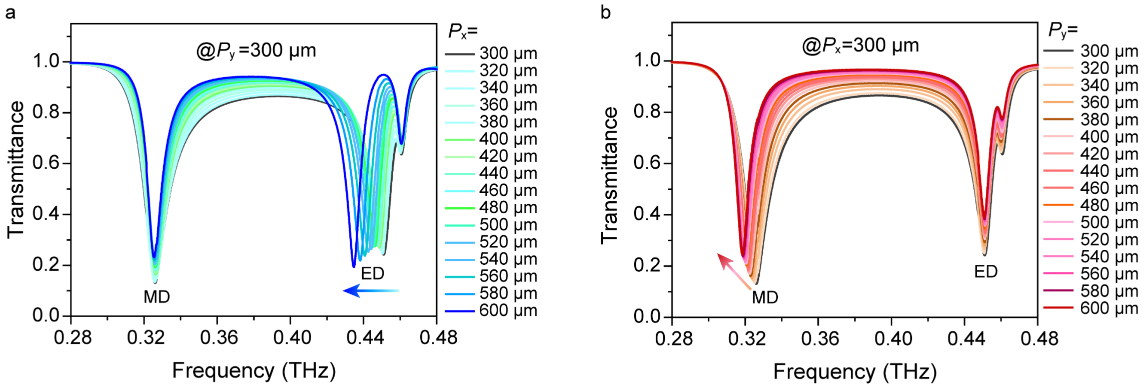

Supplementary Fig. S1. Simulation results of ED and MD resonances versus microsphere spacing (P_x and P_y) were obtained under ideal vacuum conditions. (a) for x-direction (P_x increases from 300 to 600 μm); (b) for y-direction (P_y increases from 300 to 600 μm).

Note S2. Analysis of physical mechanisms of strain-dependent THz response based on electromagnetic coupling between microspheres.

We analyze the effect of the electromagnetic near-field couplings on the Mie resonance in the ZrO_2 resonator array shown in Fig. S2. Both the impacts of the applied strain and the induced strain are incorporated in the Lagrangian analysis. However, the induced strain has a weaker impact on the near-field couplings than the applied strain since the compression due to induced strain is very limited. For example, in the ED resonance, the Lagrangian of the coupled resonator system can be expressed as

$$\begin{aligned}\mathcal{L}^{\mathrm{ED}} &= \sum_{x,y} \frac{L}{2}\left[(\ddot{Q})^2_{x,y} - 2\kappa^{\mathrm{ED}}_{\mathrm{HT}}(\ddot{Q})_{x,y}(\ddot{Q})_{x+1,y} + 2\kappa^{\mathrm{ED}}_{\mathrm{HL}}(\ddot{Q})_{x,y}(\ddot{Q})_{x,y+1}\right] \\ &- \frac{1}{2C}\left[(Q)^2_{x,y} - 2\kappa^{\mathrm{ED}}_{\mathrm{EL}}(Q)_{x,y}(Q)_{x+1,y} + 2\kappa^{\mathrm{ED}}_{\mathrm{ET}}(Q)_{x,y}(Q)_{x,y+1}\right]\end{aligned} \quad . \tag{17}$$

The coupling effects described in above equation only take the four nearest neighboring resonators, which are marked with red circles, as shown in Fig. S2 The subscripts for the coupling coefficients $\kappa^{\mathrm{ED}}_{\mathrm{HT}}$, $\kappa^{\mathrm{ED}}_{\mathrm{HL}}$, $\kappa^{\mathrm{ED}}_{\mathrm{EL}}$, and $\kappa^{\mathrm{ED}}_{\mathrm{ET}}$ denote the transverse (T) and longitudinal (L) coupling strengths of the magnetic (*h*) and electric (*e*) fields. Subsequently, by solving the Euler–Lagrange equations[S24]

$$\frac{\partial}{\partial t}\left(\frac{\partial \mathcal{L}^{\mathrm{ED}}}{\partial \dot{Q}_{\mathrm{d}}}\right) = \frac{\partial \mathcal{L}^{\mathrm{ED}}}{\partial Q_{\mathrm{d}}}, \tag{18}$$

we arrive at

$$\left[(\ddot{Q}_{\mathrm{d}})^2_{x,y} - \kappa^{\mathrm{ED}}_{\mathrm{HT}}(\ddot{Q}_{\mathrm{d}})_{x+1,y} + \kappa^{\mathrm{ED}}_{\mathrm{HL}}(\ddot{Q}_{\mathrm{d}})_{x,y+1}\right] + \frac{1}{LC}\left[(Q_{\mathrm{d}})^2_{x,y} - \kappa^{\mathrm{ED}}_{\mathrm{EL}}(Q_{\mathrm{d}})_{x+1,y} + \kappa^{\mathrm{ED}}_{\mathrm{ET}}(Q_{\mathrm{d}})_{x,y+1}\right] = 0 \tag{19}$$

Since the plane-wave excitation is uniform across the array, the coupled dielectric resonator array operates in the symmetrical mode. Thus, all the resonators have the same charge distributions, and they oscillate collectively in phase, yielding $(Q_{\mathrm{d}})_{x,y} = (Q_{\mathrm{d}})_{x+1,y} = (Q_{\mathrm{d}})_{x,y+1}$ and $(\ddot{Q}_{\mathrm{d}})_{x,y} = (\ddot{Q}_{\mathrm{d}})_{x+1,y} = (\ddot{Q}_{\mathrm{d}})_{x,y+1}$. Solving Eq. (19) yields the ED ransonance eigenfrequency of the coupled ZrO_2 array can be obtained as

$$f_s^{\mathrm{ED}} = f_0^{\mathrm{ED}}\sqrt{\frac{1+\kappa^{\mathrm{ED}}_{\mathrm{ET}} - \kappa^{\mathrm{ED}}_{\mathrm{EL}}}{1+\kappa^{\mathrm{ED}}_{\mathrm{HL}} - \kappa^{\mathrm{ED}}_{\mathrm{HT}}}}, \tag{20}$$

where the decoupled resonance frequency is f_0. By referring to the near-field expressions for the infinitesimal electric dipole, these coefficients can be linked to the unit cell sizes P_x and P_y as well as the wavenumber *k* in the PDMS via[S21]

$$\kappa^{\mathrm{ED}}_{\mathrm{HT}} = h^{\mathrm{ED}}_{\mathrm{T}} / (kP_x^3)\,;\, \kappa^{\mathrm{ED}}_{\mathrm{HL}} = h^{\mathrm{ED}}_{\mathrm{L}} / (kP_y^3)$$

$$\boldsymbol{\kappa}_{\mathrm{ET}}^{\mathrm{ED}} = e_{\mathrm{T}}^{\mathrm{ED}} / P_{\mathrm{y}}^{2}\,; \boldsymbol{\kappa}_{\mathrm{EL}}^{\mathrm{ED}} = e_{\mathrm{L}}^{\mathrm{ED}} / P_{x}^{2} \tag{21}$$

The constants $h_{\mathrm{T}}^{\mathrm{ED}}$, $h_{\mathrm{L}}^{\mathrm{ED}}$, $e_{\mathrm{T}}^{\mathrm{ED}}$, and $e_{\mathrm{L}}^{\mathrm{ED}}$ represent the effects of an oscillating current source radiating as an electric dipole in the near-field along transverse and longitudinal directions. While in the electric dipole coupling species, the magnetic field effect is very weak ($h_{\mathrm{T}}^{\mathrm{ED}}$, $h_{\mathrm{L}}^{\mathrm{ED}}$ ≈0), so Eq. (20) can be further simplified to

$$f_{s}^{\mathrm{ED}} \approx f_{0}^{\mathrm{ED}} \sqrt{1 + \kappa_{\mathrm{ET}}^{\mathrm{ED}} - \kappa_{\mathrm{EL}}^{\mathrm{ED}}} \tag{22}$$

Similarly, in magnetic dipole coupling, the electric field effect is very weak ($e_{\mathrm{T}}^{\mathrm{MD}}$, $e_{\mathrm{L}}^{\mathrm{MD}}$≈0), so the MD coupled resonance frequency can be simplified to

$$f_{s}^{\mathrm{MD}} \approx f_{0}^{\mathrm{MD}} \sqrt{\frac{1}{1 + \kappa_{\mathrm{HL}}^{\mathrm{MD}} - \kappa_{\mathrm{HT}}^{\mathrm{MD}}}}\,. \tag{23}$$

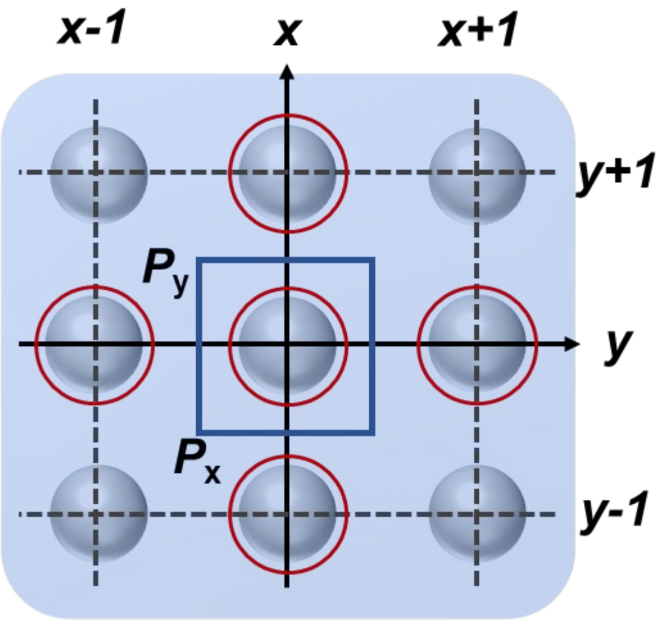

Supplementary Fig. S2. Illustration of near-field coupling among four neighboring ZrO_2-microsphere resonators.

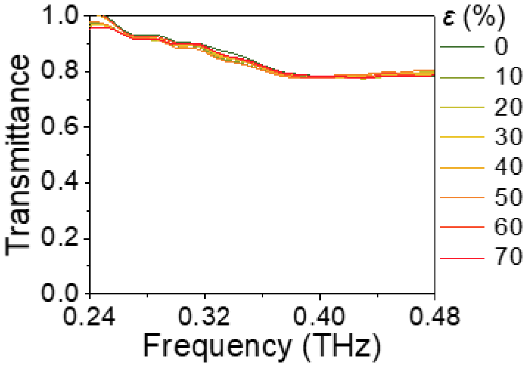

Supplementary Fig. S3. Measured transmission spectra of a pure PDMS with the identical thickness to our device under different external strains. The pure PDMS film shows high and stable transmittance even under stretchable state, consisting well with its intrinsic material property.

Note S3. The effect of PDMS on ED and MD resonances.

To uncover the effect of the substrate PDMS on the ED and MD resonances, we introduced PDMS into the initial ZrO_2 microsphere array (Fig. 2c of the main text, and Fig. S4a) for simulation that was used for establishing the basic sensing mechanism. As shown in Fig. S15b, the PDMS is gradually added towards two opposite directions from the reference plane (dot line shown in Fig. S4b) at the center of the ZrO_2 sphere. When Δh increases from 0 to 260 μm, three stages can be observed from the ED and MD resonance shift.

Stage 1: the ED and MD resonances appear large shift (Fig. S4c), when the PDMS increases from 0 to wrapping the ZrO_2 sphere just right (namely Δh is equal to 80 μm). During this process, the MD resonance gradually moves to higher frequency while the ED one shifts to the opposite direction, which can be attributed by the decayed ED and MD amplitude of the ZrO_2 microsphere array in the PDMS substrate (Fig. S4g) compared to that in the vacuum background (Fig. S4f).

Stage 2: due to the little variation in E-field and H-field distribution (Figs. S4g and h), the ED resonance only appears fine shift (Fig. S4c) while the MD resonance is almost fixed, when the PDMS increases from 80 to 100 μm.

Stage 3: when Δh is larger than 100 μm, the ED and MD resonance frequency will keep stable (Fig. S4e) as a result of the almost same E-field and H-field distributions (Figs. S4h and i)). In addition, with the increase of Δh, more THz energy can be consumed through the dielectric loss of the PDMS substrate. So that, fewer E-field can be observed localized in the PDMS substrate (white wireframes in Figs. S4h-i). That's the reason why the transmittance at the ED resonance gradually decays as Δh increases.

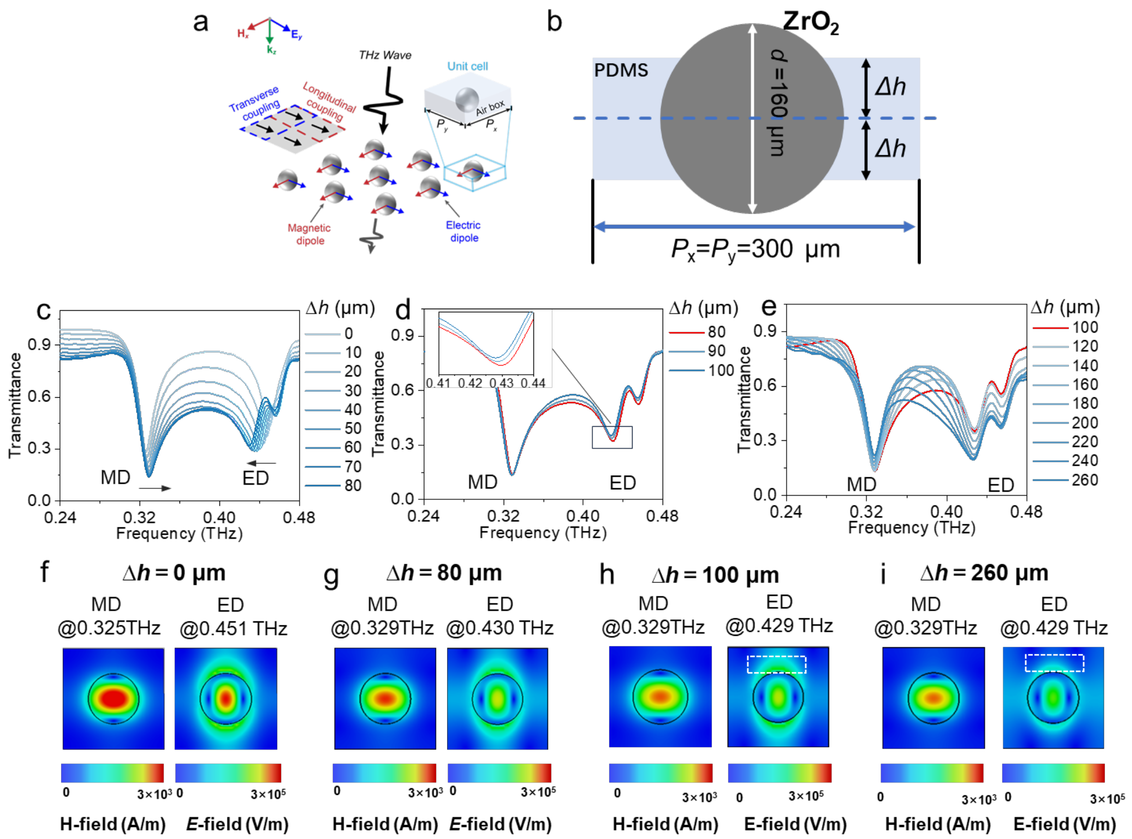

Supplementary Fig. S4. Effect of the PDMS on ED and MD resonances. (a) Schematic diagram of the ZrO_2 microsphere array in the vacuum. (b) Schematic for analyzing the effect on the ED and MD resonances. (c-e) Simulated transmittance spectra of the meta-sensor in terms of different *Δh*. Simulated E-field and H-field distributions across the center of the microspheres: (f) for *Δh* = 0 μm; (g) for *Δh* = 80 μm; (h) for *Δh* = 100 μm; (i) for *Δh* = 260 μm.

Note S4. Fabrication of the flexible strain meta-sensor array with self-cleaning capability. As shown in Fig. S5a for the fabrication process of the meta-sensor array. Initially, a porous template with a hexagonal distribution of holes is produced by lithography. Specifically, the hole diameter is 5 μm, the hole depth is 5 μm, and the holes are 10 μm apart from each other. The liquid PDMS after centrifugation and defoaming was then scraped and coated on the above porous template to obtain a liquid film of about 80 μm thick. Then, it was placed in a vacuum oven at 70 °C for about 10 minutes. At this stage, although the liquid PDMS has been cured, the surface still retains a specific viscosity. Next, the screen-printing template is attached to the PDMS surface and microspheres are placed on it. The microspheres fall into the holes under the thrust of the soft brush and their own gravity, forming an array of microspheres. Finally, an integrated stretchable meta-sensor was obtained by encapsulating it with liquid PDMS and curing it at 70 °C for ~ 25 minutes in a vacuum oven. The cross-sectional view of the resulting meta-sensor is shown in Fig. S5b, which clearly shows the surface superhydrophobic layer, and the array of ZrO_2 microspheres embedded in the PDMS matrix. The superhydrophobic layer is obtained by the shape of the porous template. The morphology of the sample stripped from the porous template is a column array with narrow top and wide bottom (upper right of Fig. S5b). After flame treating the sample surface for 3~10 s, secondary nanostructures can be formed on the surface of the columnar structure (lower right of Fig. S5b). It is the result of the aggregation of the organosilicon compound in the PDMS matrix on the surface, which can enhance the superhydrophobicity of the sample surface[S25,S26]. In this method described above, the processing area also dependent on the area of the employed template. By using a large-scale template, one can obtain an ultra-large-scale meta-sensor array, which is highly desirable in practical applications.

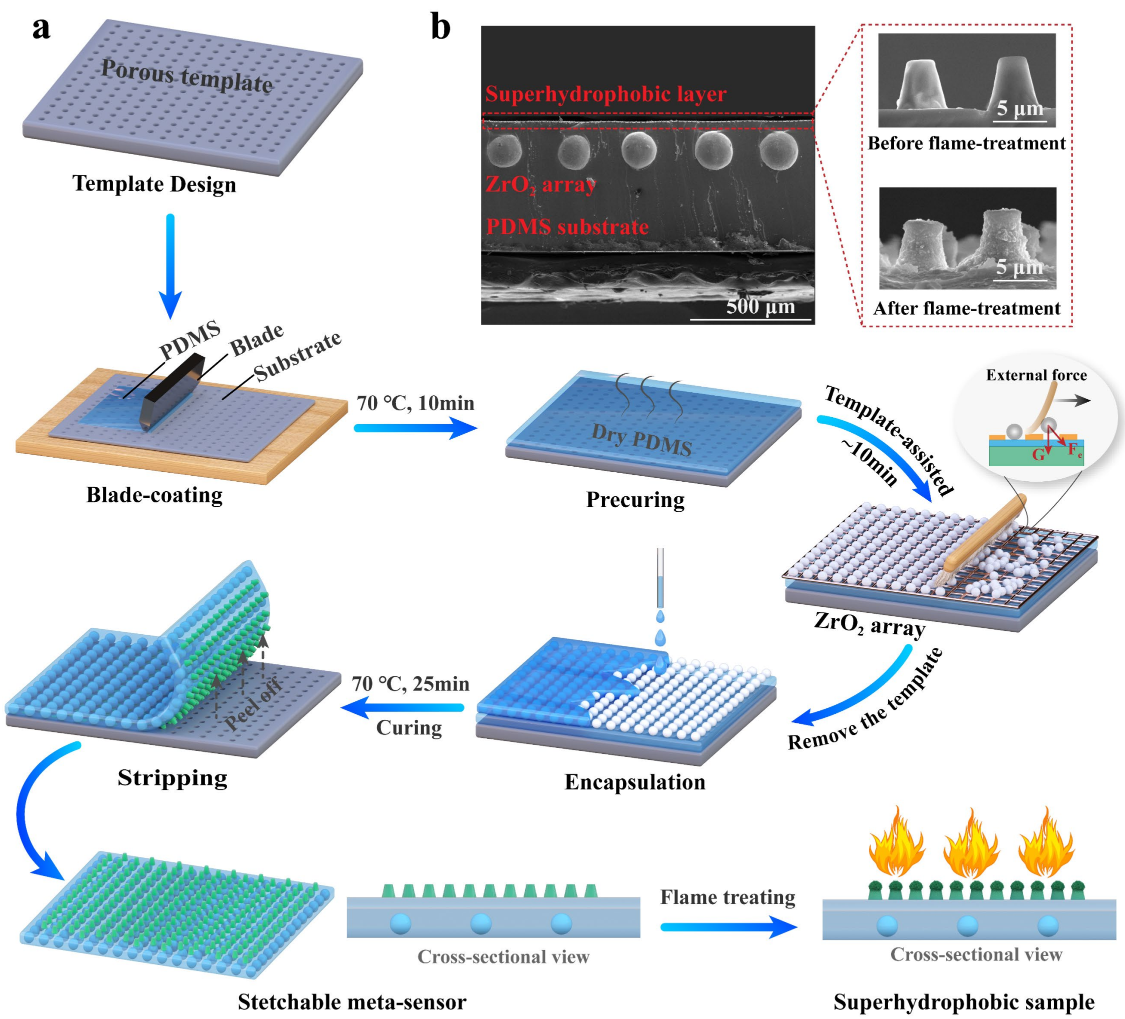

Supplementary Fig. S5. Fabrication process of the flexible strain meta-sensor array. (a) Schematic illustration of the fabrication process of stretchable meta-sensor array with self-cleaning effect. (b) SEM image of the section of the meta-sensor, in which the changes of the top hydrophobic layer before and after flame treatment can be seen in the inset on the right.

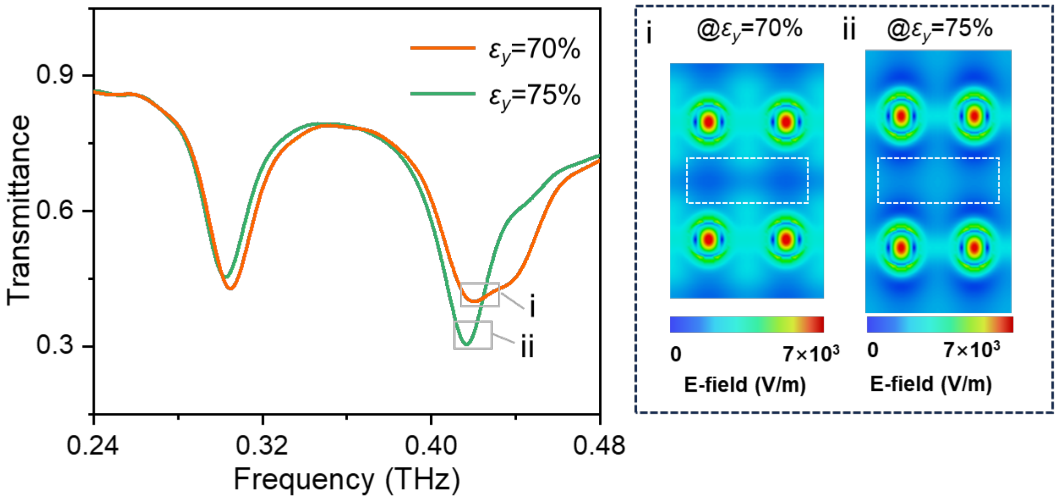

Supplementary Fig. S6. Simulated transmission spectra of meta-sensor arrays at 70% and 75% strain, and E-field distributions (@ the corresponding ED resonances) across the centre *x*-*y* plane of the microspheres as ε_y=70% (i) and ε_y=75% (ii). When the *y*-directional strain is larger than 70%, the originally stable ED resonance related to the *x*-directional strain will shift to lower frequency effecting the system calibration. This phenomenon can be attributed to the significant enhancement of the longitudinal coupling between the adjacent EDs by comparing the E-field distribution inside the white frames in insets i and ii. Therefore, the strain detection limit along *y* direction can be determined as 70%.

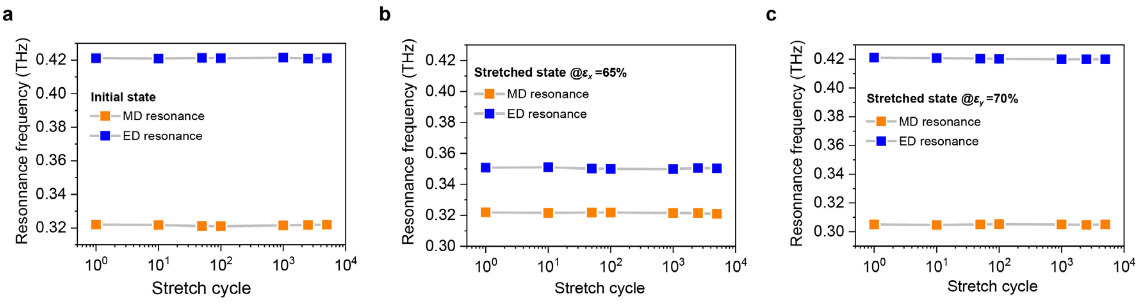

Supplementary Fig. S7. Measured ED and MD resonance frequencies of the fabricated sample after 1, 10, 50, 100, 1000, 2000 and 5000 cycles of stretching (the applied strain repeatedly varies from 0 to ~65% @ x-direction and from 0 to ~70% @ y-direction): (a) for initial state (0% strain); (b) for ~65% strain along x-direction; (c) for ~70% strain along y-direction.

Note S5. Analysis of the minimum detectable strain variations of our device.

Considering that the simulated strain-resonance frequency curves (Figs. 3f-g of the main text) are monotonically smooth and continuous, our device theoretically has the capability to detect an ultra-small strain variation. However, in practice, confined by the spectral resolution (0.001 THz) of the THz time-domain spectroscopy (TDS) device (QT-TRS1000, Quenda, China), the smallest strain variation detection performance of our device is limited.

In principle, the smallest strain variation corresponds to the point on the curves with the steepest slope. Regarding the *x*-directional strain (inset ii of Fig. 3f of the main text), the slope reaches its maximum value when the applied strain is equal to 65%. Conversely, strain-resonance frequency curve in inset i of Fig. 3g of the main text has the greatest slop with the externally *y*-directional strain of 0%. In the meantime, considering the spectral resolution of the TDS system (0.001 THz), the theoretically smallest strain variation value (0.63% @ *x* direction and 2.7% @ *y* direction) can be successfully obtained through shifting the resonance frequency by 0.001 THz towards the lower slop direction as shown in the insets of Figs. 3f-g. Then the corresponding test under the strain condition obtained above was conducted to observe the resonance frequency shifting. From Figs. 3i-j of the main text, ~0.001 THz resonance frequency shifting can be implemented in the experiment, which is consistent with the simulated predictions (inset ii of Fig. 3f and inset i of Fig. 3g of the main text). We statistically analyze the above measured results. Trough 100-round test (Fig. S8), the average resonance frequency and the standard deviation can be calculated as [0.3511 THz/0.0002 THz @ 64.37% strain along *x* direction; 0.3500 THz/0.0004 THz @ 65% strain along *x* direction] (Fig. S8a) and [0.3221 THz/0.0004 THz @ 0 % strain along y direction; 0.3210 THz/0.0005 THz @ 2.7% strain along *y* direction] (Fig. S8b). Therefore, the smallest strain variation that can be detected by our design was finalized as 0.63% @ *x* direction and 2.7% @ *y* direction. Their difference can be attributed to the discrepancy of the corresponding resonance frequency shift bandwidth.

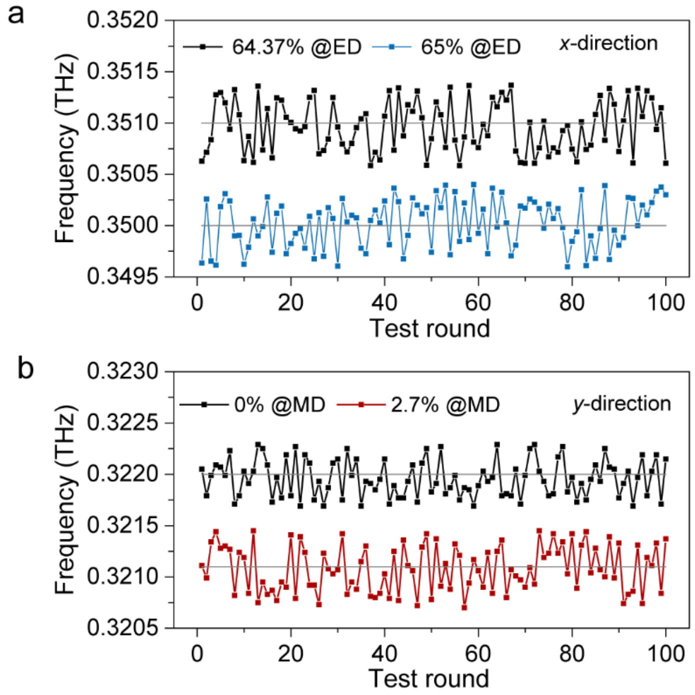

Supplementary Fig. S8. Statistical analysis for the minimum strain variations that can be detected by our device: (a) for *x*-directional strain; (b) for *y*-directional strain.

Note S6. Calculation of the GF of our device.

By analogy, we define the ratio of the relative frequency shift of the resonance peak to the strain as the GF of our device as follows

$$\mathrm{GF}=\frac{\Delta f}{f_0 \Delta \varepsilon} \tag{24}$$

where Δf is the resonance frequency shift value from the initial resonance frequency f_0, and $\Delta\varepsilon$ denotes the applied strain. Then, by substituting the measured results (Figs. 3f-g in the main text) into Eq. (24), strain-$\Delta f/f_0$ curves and the corresponding fitted ones can be obtained as shown in Fig. 3k of the main text. Following, the maximum slopes of the fitted curves, which correspond to the maximum GF, are calculated to be ~0.413 @ x direction and ~0.09 @ y direction.

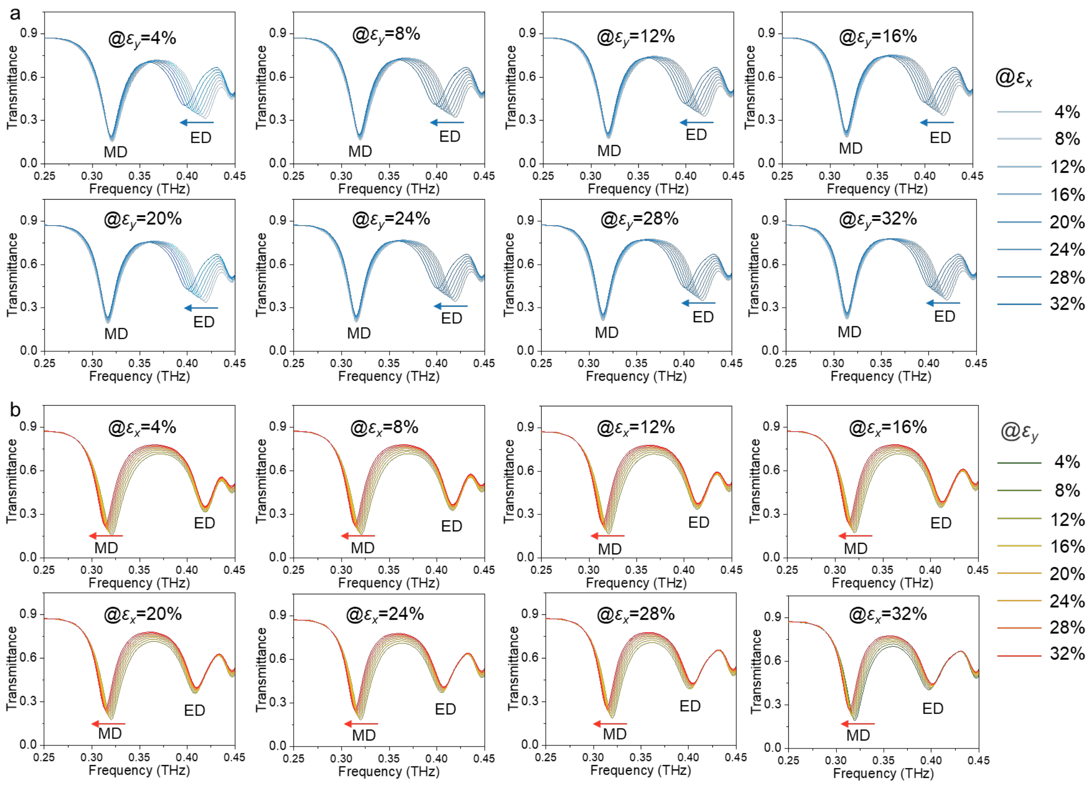

Supplementary Fig. S9. Simulated transmittance spectra of the meta-sensor array simultaneously applied with *x*- and *y*-directional tensile strain: (a) *x*-directional tensile strain varying from 4% to 32% while the orthometric strain is maintained at 4%, 8%, 12%, 16%, 20%, 24%, 28% and 32%; (b) *y*-directional tensile strain varying from 4% to 32% while the orthometric strain is maintained at 4%, 8%, 12%, 16%, 20%, 24%, 28% and 32%.

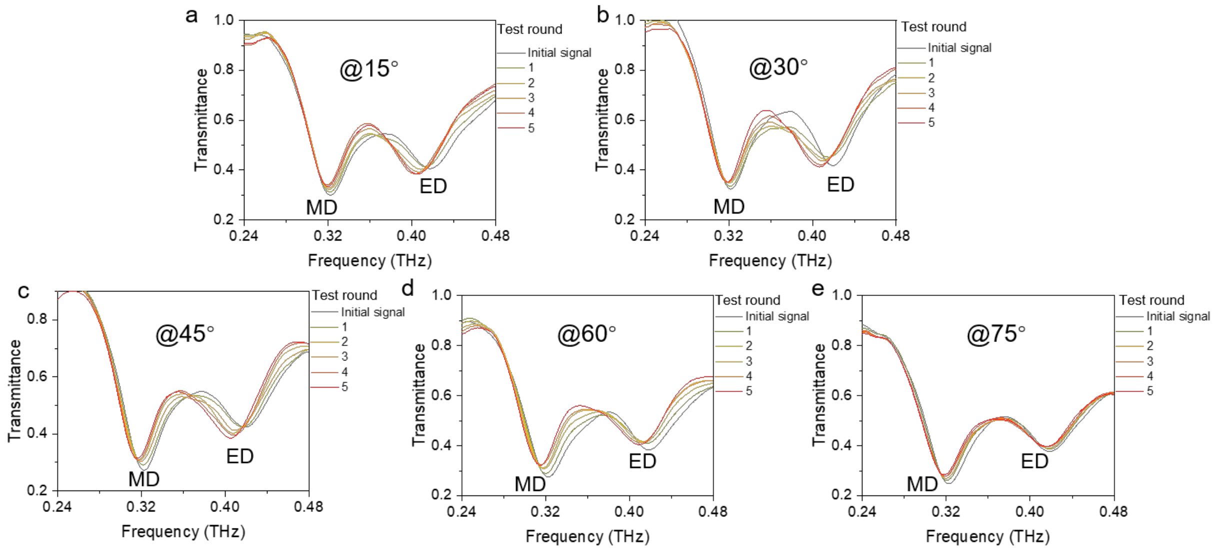

Supplementary Fig. S10. Meassured THz transmission spectra of the sample stretched along different angles: (a) 15°; (b) 30°; (c) 45°; (d) 60°; (e) 75°.

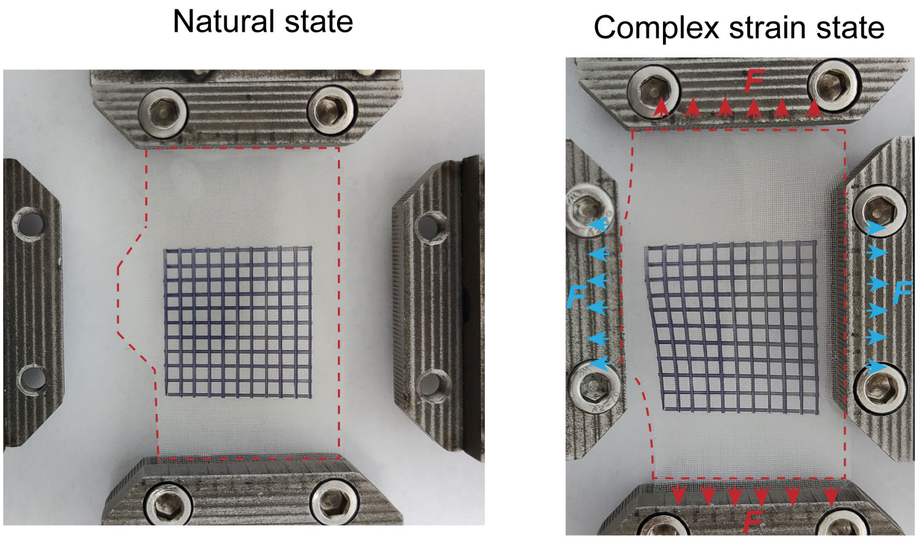

Supplementary Fig. S11. Demonstration of the bi-directional tensile test. The sample can be clamped by four ends of the fixtures, through the movement of the screw to strain the sample. (a) The original state of the sample when no strain occurs. (b) The sample is stretched first along the y-direction and then along the x-direction, showing a non-uniform strain state. The meta-sensor array is shown within the red dashed squares and the red and blue arrows indicate the tension applied in the y- and x- directions respectively.

Table S2. Measured MD and ED resonance information across the 10 × 10 girds on the sample surface by 2D strain mapping.

Position	@MD	@ED	Position	@MD	@ED	Position	@MD	@ED	Position	@MD	@ED
(1,10)	0.3187	0.4100	(6,8)	0.3176	0.4166	(1,5)	0.3190	0.4131	(6,3)	0.3180	0.4160
(2,10)	0.3182	0.4117	(7,8)	0.3180	0.4165	(2,5)	0.3192	0.4130	(7,3)	0.3184	0.4156
(3,10)	0.3186	0.4082	(8,8)	0.3187	0.4165	(3,5)	0.3196	0.4143	(8,3)	0.3175	0.4161
(4,10)	0.3180	0.4104	(9,8)	0.3175	0.4168	(4,5)	0.3197	0.4200	(9,3)	0.3165	0.4120
(5,10)	0.3171	0.4147	(10,8)	0.3154	0.4162	(5,5)	0.3195	0.4188	(10,3)	0.3178	0.4156
(6,10)	0.3186	0.4125	(1,7)	0.3183	0.4103	(6,5)	0.3177	0.4138	(1,2)	0.3168	0.4155
(7,10)	0.3170	0.4152	(2,7)	0.3180	0.4114	(7,5)	0.3176	0.4127	(2,2)	0.3171	0.4120
(8,10)	0.3163	0.4183	(3,7)	0.3185	0.4145	(8,5)	0.3181	0.4120	(3,2)	0.3180	0.4138
(9,10)	0.3165	0.4146	(4,7)	0.3193	0.4156	(9,5)	0.3165	0.4158	(4,2)	0.3169	0.4138
(10,10)	0.3168	0.4190	(5,7)	0.3191	0.4122	(10,5)	0.3153	0.4117	(5,2)	0.3180	0.4124
(1,9)	0.3172	0.4030	(6,7)	0.3187	0.4160	(1,4)	0.3186	0.4167	(6,2)	0.3174	0.4180
(2,9)	0.3180	0.4068	(7,7)	0.3175	0.4200	(2,4)	0.3174	0.4140	(7,2)	0.3188	0.4187
(3,9)	0.3188	0.4100	(8,7)	0.3175	0.4200	(3,4)	0.3183	0.4153	(8,2)	0.3178	0.4140
(4,9)	0.3195	0.4140	(9,7)	0.3159	0.4144	(4,4)	0.3188	0.4160	(9,2)	0.3169	0.4129
(5,9)	0.3185	0.4139	(10,7)	0.3158	0.4126	(5,4)	0.3196	0.4161	(10,2)	0.3193	0.4140
(6,9)	0.3170	0.4108	(1,6)	0.3180	0.4087	(6,4)	0.3191	0.4162	(1,1)	0.3150	0.4130
(7,9)	0.3180	0.4161	(2,6)	0.3186	0.4109	(7,4)	0.3180	0.4138	(2,1)	0.3175	0.4160
(8,9)	0.3184	0.4169	(3,6)	0.3190	0.4153	(8,4)	0.3171	0.4129	(3,1)	0.3177	0.4170
(9,9)	0.3160	0.4153	(4,6)	0.3200	0.4139	(9,4)	0.3170	0.4096	(4,1)	0.3169	0.4134
(10,9)	0.3154	0.4169	(5,6)	0.3183	0.4162	(10,4)	0.3180	0.4105	(5,1)	0.3160	0.4150
(1,8)	0.3185	0.4074	(6,6)	0.3175	0.4190	(1,3)	0.3160	0.4178	(6,1)	0.3143	0.4156
(2,8)	0.3190	0.4096	(7,6)	0.3188	0.4199	(2,3)	0.3165	0.4194	(7,1)	0.3176	0.4192
(3,8)	0.3190	0.4058	(8,6)	0.3182	0.4196	(3,3)	0.3175	0.4160	(8,1)	0.3190	0.4187
(4,8)	0.3196	0.4170	(9,6)	0.3168	0.4153	(4,3)	0.3170	0.4168	(9,1)	0.3187	0.4172
(5,8)	0.3190	0.4155	(10,6)	0.3140	0.4130	(5,3)	0.3173	0.4177	(10,1)	0.3187	0.4156

Table S3. Calculation of the strain values across the 10 × 10 girds on the sample surface by means of visual computational analysis (original length and the length after strain of the mesh for *y* direction).

y-direction											
Position	Natural state/ mm	Complex strain state/ mm	Position	Natural state/mm	Complex strain state/ mm	Position	Natural state/mm	Complex strain state/mm	Position	Natural state/mm	Complex strain state/mm
(1,10)	3.080	3.400	(6,8)	3.119	3.587	(1,5)	3.049	3.367	(6,3)	3.037	3.455
(2,10)	3.116	3.518	(7,8)	3.157	3.581	(2,5)	3.049	3.369	(7,3)	3.123	3.503
(3,10)	3.100	3.474	(8,8)	3.139	3.532	(3,5)	3.080	3.352	(8,3)	3.103	3.564
(4,10)	3.080	3.482	(9,8)	3.172	3.671	(4,5)	3.080	3.335	(9,3)	3.029	3.579
(5,10)	3.100	3.632	(10,8)	3.119	3.783	(5,5)	3.126	3.399	(10,3)	3.089	3.512
(6,10)	3.172	3.552	(1,7)	2.752	3.084	(6,5)	3.042	3.474	(1,2)	3.080	3.649
(7,10)	3.177	3.718	(2,7)	2.781	3.169	(7,5)	3.023	3.496	(2,2)	3.129	3.655
(8,10)	3.139	3.754	(3,7)	2.806	3.171	(8,5)	3.126	3.546	(3,2)	3.126	3.568
(9,10)	3.176	3.770	(4,7)	2.852	3.134	(9,5)	3.034	3.584	(4,2)	3.095	3.621
(10,10)	3.177	3.741	(5,7)	2.864	3.159	(10,5)	2.957	3.659	(5,2)	3.145	3.568
(1,9)	2.834	3.277	(6,7)	2.857	3.200	(1,4)	3.018	3.396	(6,2)	3.169	3.643
(2,9)	2.849	3.209	(7,7)	2.834	3.294	(2,4)	2.866	3.323	(7,2)	3.152	3.512
(3,9)	2.888	3.200	(8,7)	2.861	3.317	(3,4)	2.920	3.305	(8,2)	3.131	3.513
(4,9)	2.911	3.184	(9,7)	2.811	3.417	(4,4)	3.003	3.329	(9,2)	3.015	3.542
(5,9)	2.896	3.244	(10,7)	2.829	3.460	(5,4)	3.065	3.337	(10,2)	3.092	3.429
(6,9)	2.868	3.323	(1,6)	2.946	3.335	(6,4)	3.034	3.307	(1,1)	2.972	3.650
(7,9)	2.820	3.228	(2,6)	3.034	3.396	(7,4)	2.957	3.334	(2,1)	2.980	3.460
(8,9)	2.911	3.298	(3,6)	3.080	3.386	(8,4)	2.957	3.428	(3,1)	3.025	3.482
(9,9)	2.820	3.377	(4,6)	3.065	3.290	(9,4)	2.957	3.461	(4,1)	3.049	3.577
(10,9)	2.841	3.489	(5,6)	3.046	3.404	(10,4)	2.957	3.374	(5,1)	2.985	3.618
(1,8)	3.100	3.446	(6,6)	3.032	3.514	(1,3)	2.972	3.558	(6,1)	2.972	3.745
(2,8)	3.109	3.432	(7,6)	3.080	3.421	(2,3)	3.002	3.552	(7,1)	3.109	3.594
(3,8)	3.066	3.407	(8,6)	3.160	3.555	(3,3)	3.029	3.478	(8,1)	3.119	3.454
(4,8)	3.116	3.400	(9,6)	3.080	3.629	(4,3)	3.031	3.532	(9,1)	3.131	3.523
(5,8)	3.119	3.452	(10,6)	3.080	3.986	(5,3)	3.063	3.546	(10,1)	3.219	3.603

Table S4. Calculation of the strain values across the 10 × 10 girds on the sample surface by means of visual computational analysis (original length and the length after strain of the mesh for *x* direction).

x-direction											
Position	Natural state	Complex strain state	Position	Natural state	Complex strain state	Position	Natural state	Complex strain state	Position	Natural state	Complex strain state
(1,10)	3.003	3.315	(6,8)	2.840	3.267	(1,5)	2.880	3.180	(6,3)	3.003	3.417
(2,10)	2.849	3.217	(7,8)	3.109	3.527	(2,5)	2.834	3.130	(7,3)	3.188	3.576
(3,10)	3.042	3.409	(8,8)	2.734	3.077	(3,5)	3.126	3.403	(8,3)	2.926	3.361
(4,10)	2.926	3.308	(9,8)	3.253	3.763	(4,5)	2.880	3.118	(9,3)	3.188	3.766
(5,10)	3.049	3.572	(10,8)	2.941	3.568	(5,5)	3.080	3.349	(10,3)	3.049	3.467
(6,10)	2.911	3.259	(1,7)	2.917	3.269	(6,5)	2.880	3.290	(1,2)	2.972	3.522
(7,10)	3.100	3.628	(2,7)	2.837	3.232	(7,5)	3.049	3.526	(2,2)	2.860	3.341
(8,10)	2.772	3.316	(3,7)	3.079	3.479	(8,5)	2.710	3.075	(3,2)	3.034	3.462
(9,10)	3.263	3.874	(4,7)	2.892	3.178	(9,5)	3.234	3.820	(4,2)	2.852	3.336
(10,10)	3.071	3.616	(5,7)	2.931	3.232	(10,5)	2.988	3.697	(5,2)	2.991	3.393
(1,9)	2.926	3.383	(6,7)	2.858	3.202	(1,4)	3.034	3.413	(6,2)	3.018	3.470
(2,9)	2.791	3.143	(7,7)	3.108	3.612	(2,4)	2.894	3.355	(7,2)	2.992	3.333
(3,9)	3.196	3.541	(8,7)	2.849	3.303	(3,4)	3.079	3.485	(8,2)	2.846	3.193
(4,9)	2.875	3.145	(9,7)	3.219	3.913	(4,4)	2.798	3.102	(9,2)	3.179	3.734
(5,9)	3.032	3.397	(10,7)	2.926	3.579	(5,4)	3.114	3.390	(10,2)	2.986	3.311
(6,9)	2.897	3.357	(1,6)	2.801	3.171	(6,4)	3.018	3.291	(1,1)	2.883	3.540
(7,9)	3.051	3.493	(2,6)	2.791	3.124	(7,4)	3.080	3.473	(2,1)	2.772	3.219
(8,9)	2.734	3.097	(3,6)	3.071	3.376	(8,4)	2.883	3.342	(3,1)	3.106	3.576
(9,9)	3.234	3.873	(4,6)	2.852	3.061	(9,4)	3.049	3.569	(4,1)	2.772	3.252
(10,9)	2.946	3.618	(5,6)	2.952	3.299	(10,4)	3.003	3.427	(5,1)	3.011	3.650
(1,8)	2.965	3.296	(6,6)	2.895	3.355	(1,3)	3.003	3.595	(6,1)	2.909	3.665
(2,8)	2.888	3.188	(7,6)	2.911	3.233	(2,3)	2.888	3.417	(7,1)	3.080	3.560
(3,8)	2.926	3.251	(8,6)	2.849	3.205	(3,3)	3.196	3.669	(8,1)	2.895	3.207
(4,8)	2.926	3.194	(9,6)	3.208	3.780	(4,3)	2.849	3.320	(9,1)	3.196	3.596
(5,8)	3.003	3.325	(10,6)	2.911	3.767	(5,3)	3.131	3.625	(10,1)	3.003	3.362

Note S7. Self-cleaning effect of the meta-sensor array.

For practical applications, it is essential to keep the sensor surface away from dusts, water and other interferents with strong THz adsorption. Thus self-cleaning capacity mimicing the superhydrophobic lotus leaf (Fig. S12a) was introdued to the meta-sensor array with a compatible fabrication process. Using replica method, micro pillar array was fabricated on the surface and nano organosilicon compound was further generated on the surface of the micro pillar array after flame treating for 3~10 s (Fig. S12b). Static contact angle (CA) and roll-off angle were measured from sessile water drops with a drop shape analysis instrument (DSA 100, Kruss, Hamburg) at room temperature. With the hierarchical structures and low surface energy of PDMS, the surface of the meta-sensor exhibit superhydrophobic with higher contact angles (CAs, ~163°) and smaller sliding angle (SA, ~4.5°) compared with the normal samples (S_{normal}) peeled from smooth PET (Fig. S12d, Video S1). Thus, water droplets bounce off the surface of the meta-sensor and take away the dust (with sands as a demonstration) (Figs. S12e-f, Video S2-3). These results verified the superhydrophobic and self-cleaning capacity of the fabricated meta-sensor array.

Then, the THz transmission feature of the sample with and without superhydrophobic treatment is measured considering the interference of the water and dust (Figs. S12g-h). As shown in Fig. S12g, the water covering sample (left side of Fig. S12g) absolutely loss function due to the strong THz absorption of water, while water is difficult to retain on the surface of the hydrophobically treated sample (right side of Fig. S12g)), leading to almost no influence on the transmission spectra. In contrast, the dust only decays the intensity of the resonances (left side of Fig. S12f)) and can be wiped away from the hydrophobically treated sample (right side of Fig. S12f) through flushing with the water, to keep the dynamic range of the meta-sensor. In addition, it is worth noting that the abovementioned superhydrophobic effect can be completely maintained as the strain applied to the sample gradually increases to more than 30%, denoting our design taking robust working condition adaptability (the corresponding phenomena can be found in video S4).

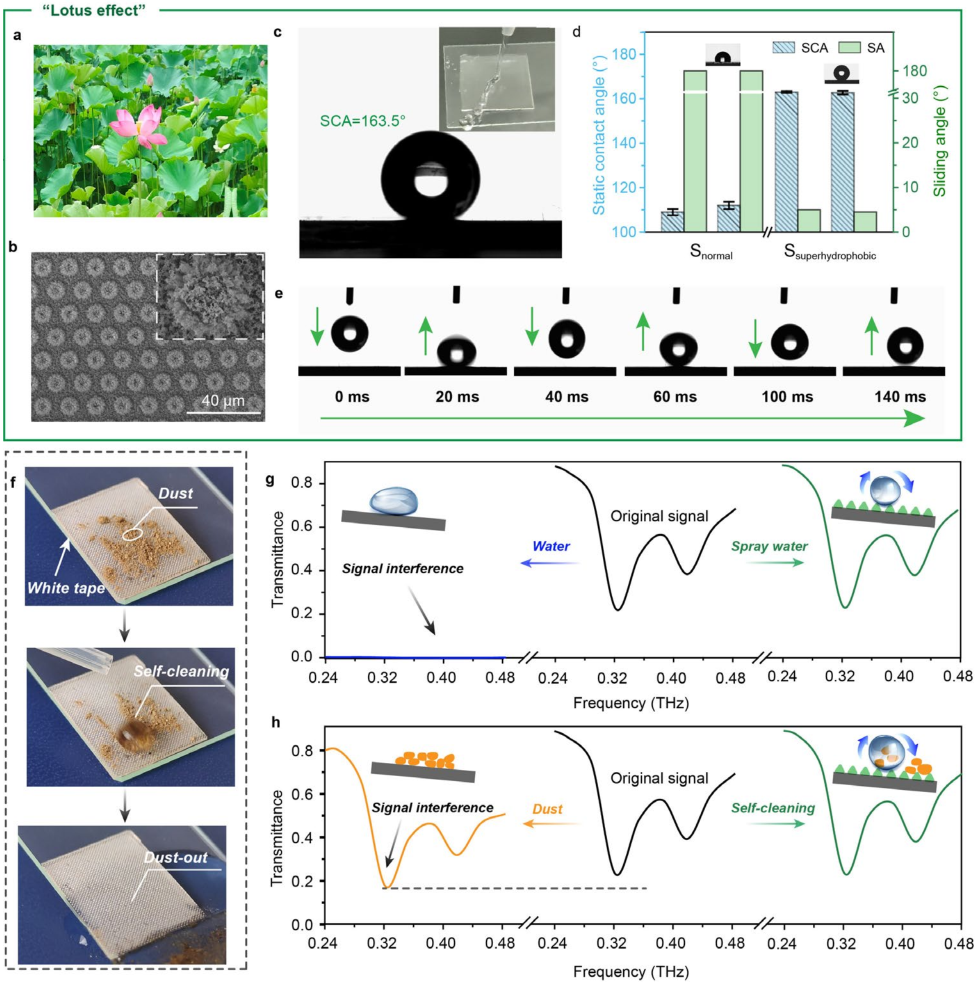

Supplementary Fig. S12. Characterization of the meta-sensor array samples with self-cleaning capacity. (a) The superhydrophobic effect of lotus leaves in nature. (b) SEM image of the micro-/nanostructure arrays on the surface of the sample after flame treatment. (c) Static water contact angle on the sample surface, where the inset shows a physical image of water droplets flowing on the sample surface. (d) Comparison of static water contact angle and sliding angle performance of normal samples (peeled from smooth PET) and superhydrophobic samples (peeled from a template and treated by a flame-treatment process). Each error bar represents the standard deviation of 6 measurements. (e) Drop dynamical impacting behaviours displayed by the selected snapshots of a droplet vertically impacting on sample surfaces. (f) Self-cleaning process on the sample surfaces with a 15° tilting angle using the dust as contaminant. (g) Comparison of the THz transmission signal of a normal sample (where the water droplet is attached to the sample surface) and a sample with a "self-cleaning effect" (where the water droplet cannot stay on the sample surface). (h) Comparison of THz transmission signals from samples with dust on the surface and after self-cleaning to remove the dust.

Note S8. Design and performance evaluation of a reflection-type meta-sensor for external strain detection.

To further verify the feasibility of the reflection-type design shown in Figs. S13a-b, we have conducted the stretching response experiments. First of all, the reflection time-domain signals of the sample applied with the unidirectional tensile strain were measured and presented in Fig. S13c (for x-directional strain) and S13d (for y-directional strain). Then, the corresponding spectra information can be obtained (Figs. S13e and h) through applying the Fourier transform to the time signals (insets in Figs. S13c-d). Similar to the transmission-type meta-sensor, when the sample is stretched along the x-direction, the measured ED resonance gradually shifts from 0.4210 THz to 0.3640 THz as the strain increases from 0 to 65% (Figs. S13e), which is consistent with the simulation results (Figs. S13f). Conversely, when the strain along the y-direction increases from 0 to 70%, the measured MD resonance (Figs. S13h) matches the simulated one (Figs. S13i), and decreases from 0.3225 THz to 0.3000 THz. Additionally, in these two processes, the remaining resonances (the MD resonance during x-direction stretching and the ED resonance during y-direction stretching) show barely shifts (Figs. S13g and j). These experimental results have successfully proved the unidirectional tensile strain detection ability of the proposed reflective meta-sensor.

In addition, bidirectional tensile strain detection performance of the proposed reflective meta-sensor has been evaluated. As shown in Fig. S14a, with the x-directional strain varying from 5% to 30% by 5% per step, the strain applied in the y-direction is fixed at 5%, 10%, 15%, 20%, 25% and 30%. In such cases, the ED resonance gradually shifts to a lower frequency as the x-directional strain increases, while the MD resonance always remains localized at a certain value only associated with the y-directional deformation ratio (5% @ 0.3212 THz, 10% @ 0.3183 THz, 15% @ 0.3165 THz, 20% @ 0.3143 THz, 25% @ 0.3126 THz, and 30% @ 0.3116 THz). By switching the strain loading, the opposite phenomenon can be revealed in Fig. S14b, i.e., the MD resonance frequency decreases with increasing strain in the y-direction, while the ED resonance always stays at a specific value, which is only related to the x-directional deformation ratio (5% @ 0.4173 THz, 10% @ 0.4148 THz, 15% @ 0.4107 THz, 20% @ 0.4072 THz, 25% @ 0.4018 THz, and 30% @ 0.3970 THz). These results demonstrate that the reflective meta-sensor also favours independent and noninterfering monitoring of the orthogonal strains. Additionally, the experimental results agree well with the simulated results shown in Figs. S15a-b, further proving the veracity of our strategy. Furthermore, in Figs. S14c-d, the dynamic ED and MD resonance frequencies during stretching have been extracted from Figs. S14a-b to directly exhibit the corresponding relation between the bidirectional strains applied to our

sensor and its resonance shifting. All the experimental results in Figs. S13 and S14 indicate that the sensing mechanism we proposed can be directly extended to the design of reflective strain sensor elements, making it more suitable for practical applications.

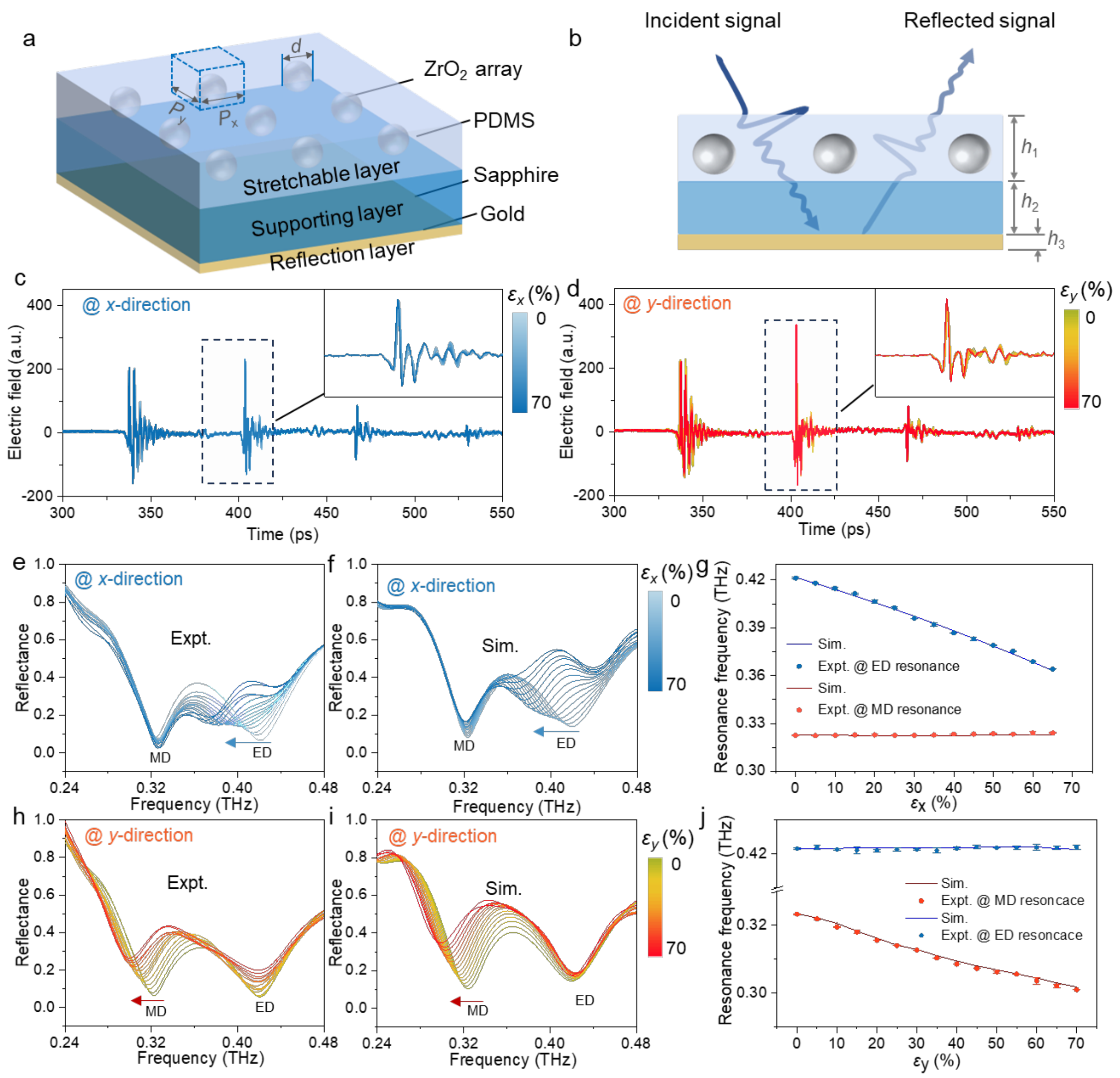

Supplementary Fig. S13. Unidirectional tensile strain detection performance evaluation of the reflective meta-sensor. (a-b) Structure diagram of reflective meta-sensor. Experimental time-domain spectra of the meta-sensor array applied with different tensile strains (varying from 0 to 70% by 5% per step): (c) for the *x* direction; (d) for the *y* direction. The structural parameters of the proposed meta-sensor are h_1 = 320 μm, h_2 = 1000 μm, h_3 = 0.2 μm, P_x = 320 μm, P_y = 320 μm, *d* = 161 μm. Corresponding frequency spectra calculated from the time-domain spectra (insets in (c) and (d)) through performing Fourier Transform: (e) for the *x* direction; (h) for the *y* direction. Simulated frequency spectra of the sample under different strains: (f) for the *x* direction; (i) for the *y* direction. Extracted resonance information from (e-f) and (h-i) are plotted in (g) and (j). The data in (g) and (j) are presented as mean ± s.d. of n ≥ 6 independent measurements.

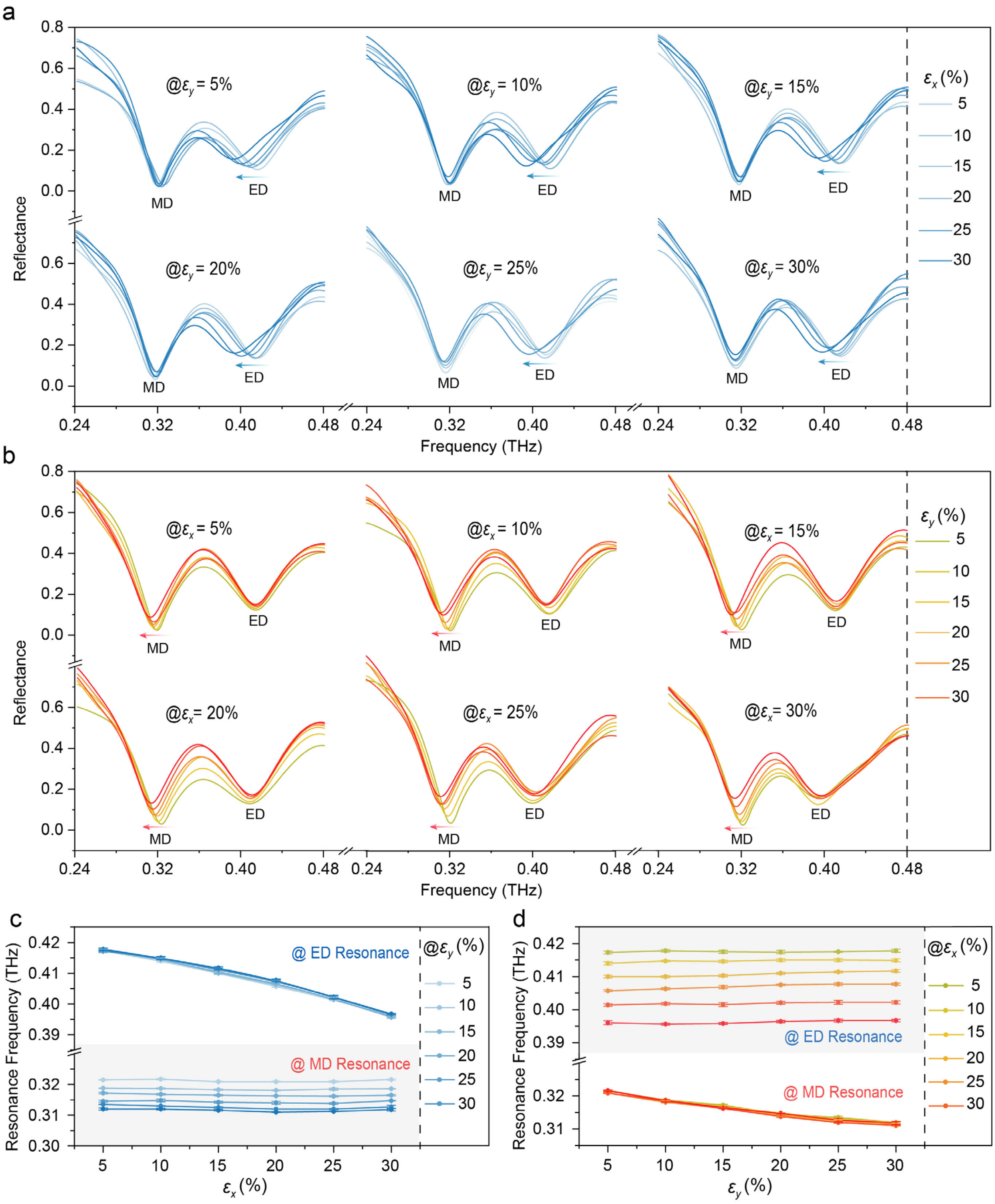

Supplementary Fig. S14. Bidirectional tensile strain detection performance evaluation of the reflective meta-sensor. Experimental reflection spectra of the meta-sensor array simultaneously applied with *x*- and *y*-directional tensile strain: (a) *x*-directional tensile strain varying from 5% to 30% while the orthometric strain is maintained at 5%, 10%, 15%, 20%, 25%, and 30%; (b) *y*-directional tensile strain varying from 5% to 30% while the orthometric strain is maintained at 5%, 10%, 15%, 20%, 25%, and 30%. Extracted resonance information from (a) and (b) are plotted in (c) and (d). The data in (c-d) are presented as mean ± s.d. of n ≥ 6 independent measurements.

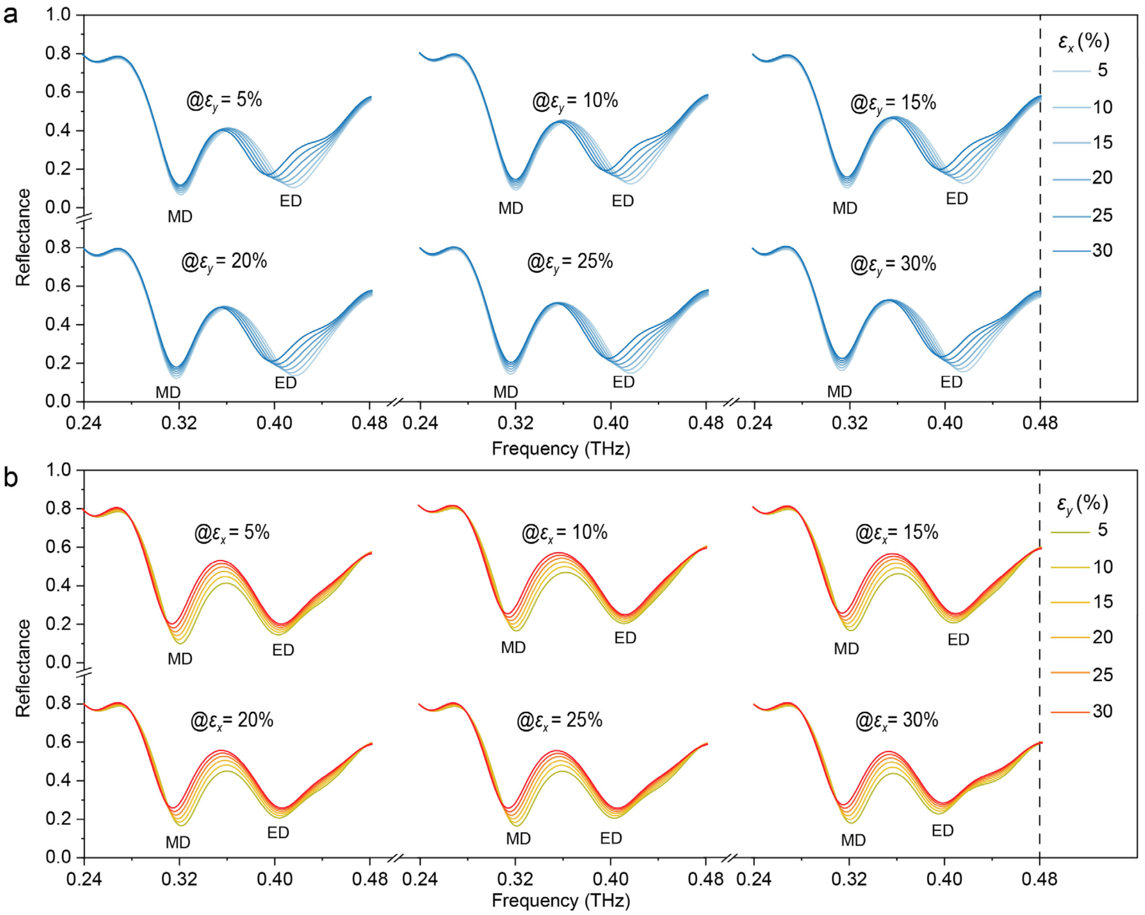

Supplementary Fig. S15. Simulated spectra of the reflective meta-sensor array simultaneously applied with *x*- and *y*-directional tensile strain: (a) *x*-directional tensile strain varying from 5% to 30% while the orthometric strain is maintained at 5%, 10%, 15%, 20%, 25%, and 30%; (b) *y*-directional tensile strain varying from 5% to 30% while the orthometric strain is maintained at 5%, 10%, 15%, 20%, 25%, and 30%.

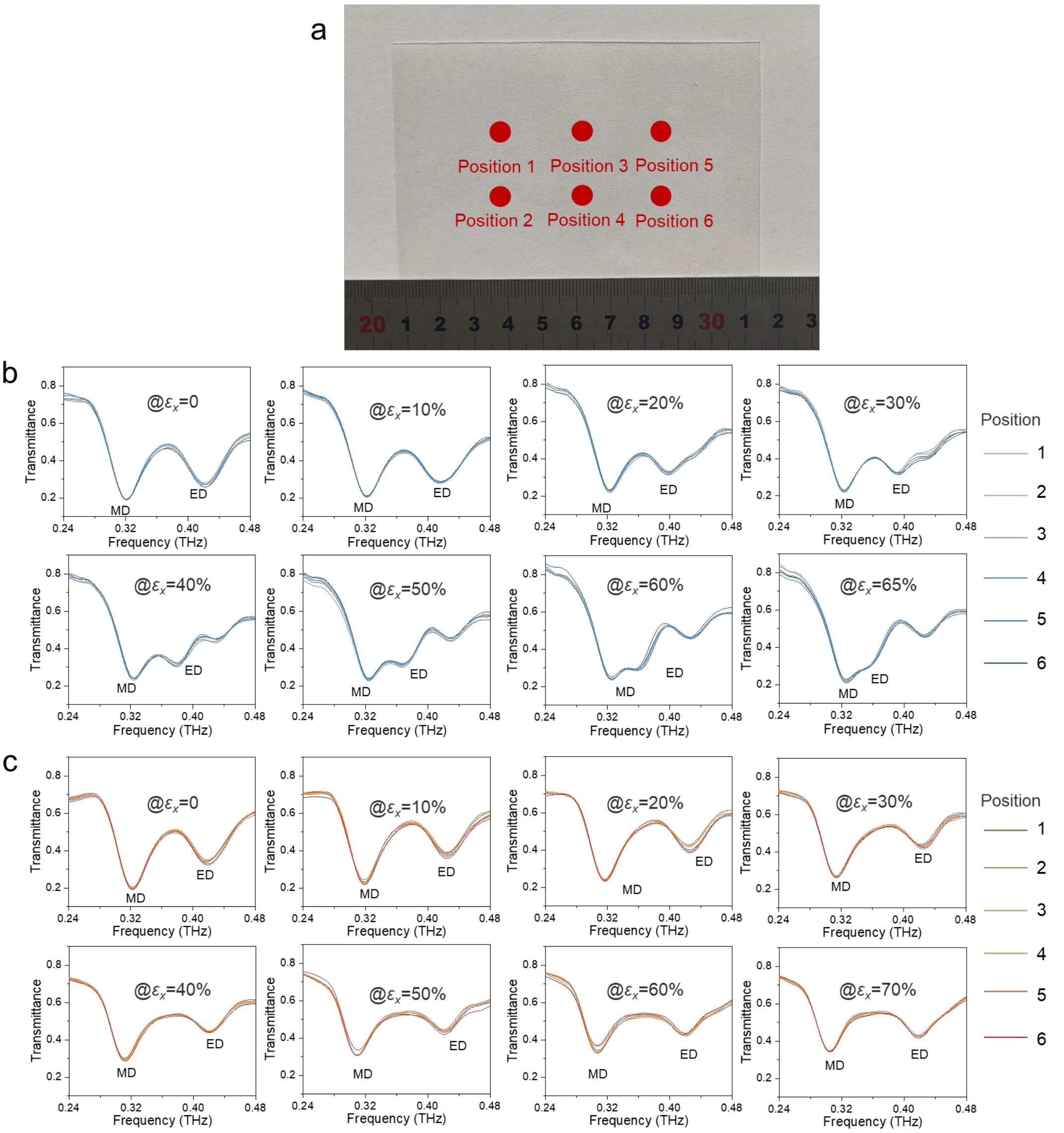

Supplementary Fig. S16. (a) Optical photographs of the sample tested with the test positions marked with red dots. Transmission spectra of the sample at different positions during stretching in the x-direction (b) and y-direction (c). We measured the resonance-strain response at the different locations of the fabricated sample (the six red points shown in a). As observed, the MD and ED resonance peaks at various positions on the sample are almost the same when subjected to the same strain in x- (b) and y-direction (c) during tensile tests.

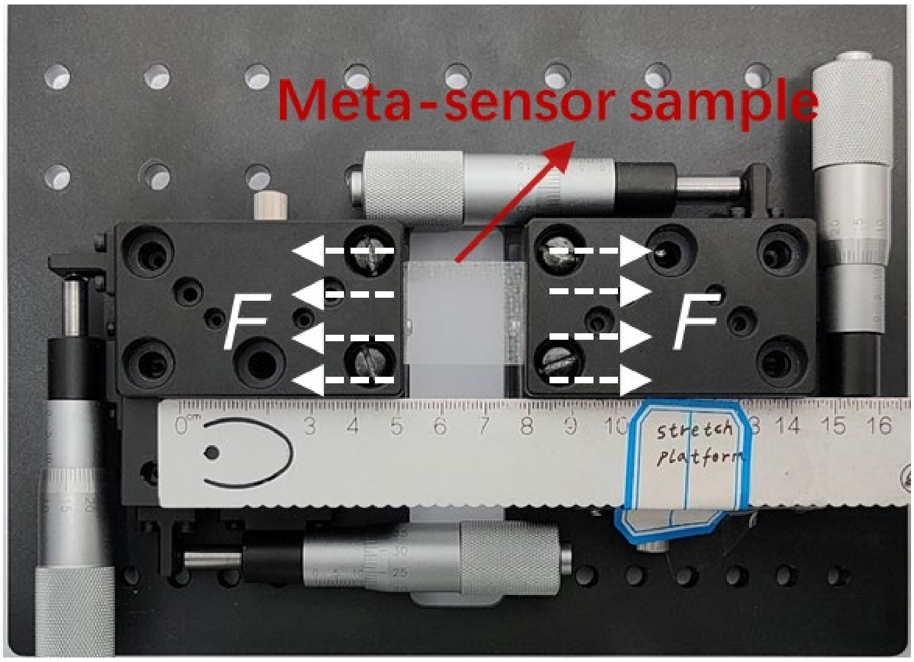

Supplementary Fig. S17. Demonstration of the unidirectional tensile test. The independent samples were fixed on the fixtures, and the fixtures were evenly moved along the *x*- or *y*-direction, respectively, and the THz transmission spectra of the samples under different strains were obtained. The mechanical strain can be produced by moving the graded micrometer screw. The size of the sample is much larger than the THz illuminate spot. Thus, although the sample is fixed on the clamp, its center is still stretched uniformly.

Note S9. Bending deformation response of the meta-sensor array.

The schematic diagram of the bending process is shown in Fig. S18a. Both ends of the sample were clamped by two movable fixtures, and then by tuning the spacing between these two fixtures, the bending degree of the sample can be modified continuously (inset of Fig. S18a). The initial length (L) of the sample without bending is 15 mm. When the sample was gradually bent along the x/y direction, little resonant frequency shifting (ED resonance @ x-directional bending; MD resonance @ y-directional bending) can be observed in Figs. S18b-c (as L decreased from 15 mm to 10 mm), denoting that our design is insensitive to the out-of-plane bending deformation.

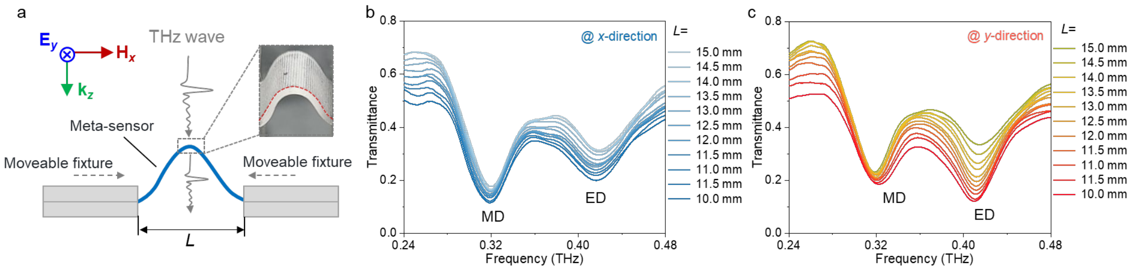

Supplementary Fig. S18. Bending response test for the meta-sensor array. (a) Schematic illustration of the bending process and the photograph of the bent meta-sensor sample. THz transmission spectrum of the meta-sensor in the process of the fixtures moving to the center: (b) for the *x*-directional bending; (c) for the *y*-directional bending.

Note S10. Twisting deformation response of the meta-sensor array.

When conduct the twisting test, as shown in Fig. S19a, one end of the sample was fixed and the other one was rotated by θ = 0°, 20°, 40°, 60°, 80°, 100°, 120°, 140°, 160°, and 180° (Fig. S19b). The measured THz spectra are presented in Fig. S19c (rotation along *x*-axis) and Fig. S19d (rotation along *y*-axis), respectively. It is easy to find that when the twisting angle gradually increases from 0° to a threshold -- 140° @ rotation along *x*-axis and 120° @ rotation along *y*-axis, both the MD and ED resonance peaks simultaneously shift towards low frequencies as well as the transmittance magnitude decays, which can be attributed to the orthogonal strains induced by the applied rotational deformation. However, once the twisting angle exceeds the corresponding thresholds, the changes in the MD and ED resonances become irregular due to the sample appears folded.

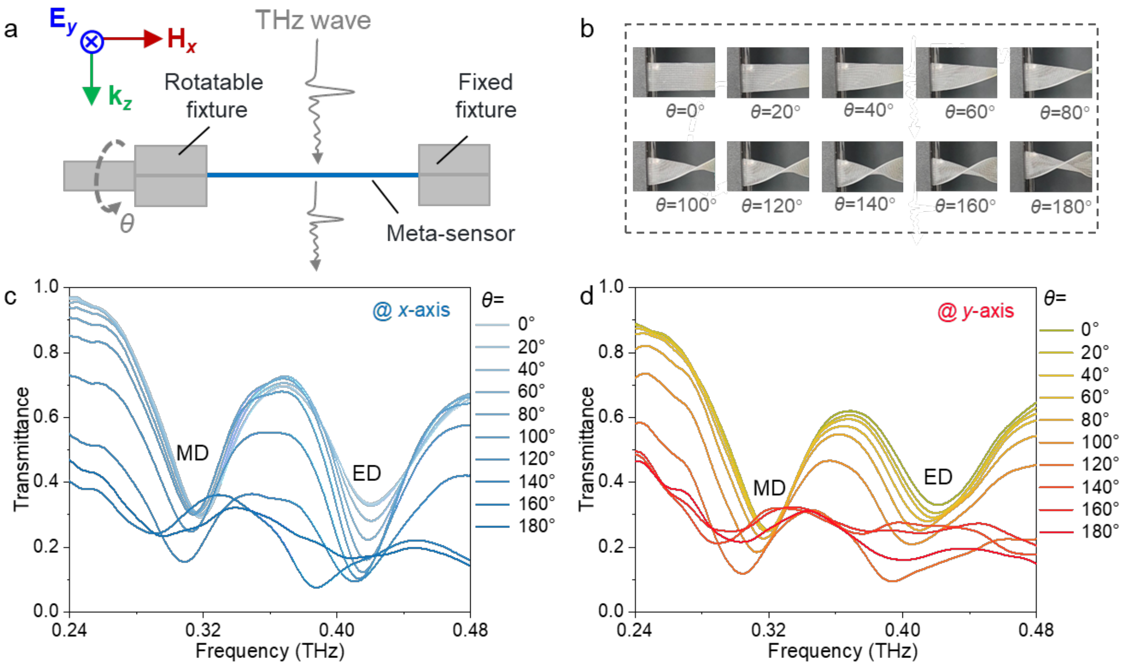

Supplementary Fig. S19. Twisting response test for the meta-sensor array. (a) Schematic illustration of the twisting process. (b) Photograph of the twisted meta-sensor. The THz transmission spectra of the meta-sensor under different twisted states: (c) for rotation along the *x*-axis; (d) for rotation along the *y*-axis.

Reference

S1. Wang, S. *et al.* Novel Safeguarding Tactile e-Skins for Monitoring Human Motion Based on SST/PDMS–AgNW–PET Hybrid Structures. *Advanced Functional Materials* **28**, 1707538 (2018).

S2. Li, M., Chen, S., Fan, B., Wu, B. & Guo, X. Printed Flexible Strain Sensor Array for Bendable Interactive Surface. *Advanced Functional Materials* **30**, 2003214 (2020).

S3. Kim, K. K. *et al.* Highly Sensitive and Stretchable Multidimensional Strain Sensor with Prestrained Anisotropic Metal Nanowire Percolation Networks. *Nano Letter* **15**, 5240–5247 (2015).

S4. Gao, Y. *et al.* Laser Direct Writing of Ultrahigh Sensitive SiC-Based Strain Sensor Arrays on Elastomer toward Electronic Skins. *Advanced Functional Materials* **29**, 1806786 (2019).

S5. Xiao, T. *et al.* 3D Printing of Flexible Strain Sensor Array Based on UV-Curable Multiwalled Carbon Nanotube/Elastomer Composite. *Advanced Materials Technologies* **6**, 2000745 (2021).

S6. Wang, Z. *et al.* Ultrastretchable Strain Sensors and Arrays with High Sensitivity and Linearity Based on Super Tough Conductive Hydrogels. *Chemistry of Materials* (2018) doi:10.1021/acs.chemmater.8b03999.

S7. Yin, B., Liu, X., Gao, H., Fu, T. & Yao, J. Bioinspired and bristled microparticles for ultrasensitive pressure and strain sensors. *Nature Communication* **9**, 5161 (2018).

S8. Hua, Q. *et al.* Skin-inspired highly stretchable and conformable matrix networks for multifunctional sensing. *Nature Communnication* **9**, 244 (2018).

S9. Wang, S. *et al.* Skin electronics from scalable fabrication of an intrinsically stretchable transistor array. *Nature* **555**, 83–88 (2018).

S10. Oh, J. Y. *et al.* Stretchable self-healable semiconducting polymer film for active-matrix strain-sensing array. *Science Advances* **5**, eaav3097 (2019).

S11. Wang, Y. *et al.* A durable nanomesh on-skin strain gauge for natural skin motion monitoring with minimum mechanical constraints. *Science Advances* **6**, eabb7043 (2020).

S12. Ota, S., Ando, A. & Chiba, D. A flexible giant magnetoresistive device for sensing strain direction. *Nature Electronics* **1**, 124–129 (2018).

S13. Zhao, X., Hua, Q., Yu, R., Zhang, Y. & Pan, C. Flexible, Stretchable and Wearable Multifunctional Sensor Array as Artificial Electronic Skin for Static and Dynamic Strain Mapping. *Advanced Electronic Materials* **1**, 1500142 (2015).

S14. Shintake, J., Piskarev, Y., Jeong, S. H. & Floreano, D. Ultrastretchable Strain Sensors Using Carbon Black-Filled Elastomer Composites and Comparison of Capacitive Versus Resistive Sensors. *Advanced Materials Technologies* **3**, 1700284 (2018).

S15. You, I. *et al.* Artificial multimodal receptors based on ion relaxation dynamics. *Science* **370**, 961–965 (2020).

S16. Sun, Q. *et al.* Active Matrix Electronic Skin Strain Sensor Based on Piezopotential-Powered Graphene Transistors. *Advanced Materials* **27**, 3411–3417 (2015).

S17. Tian, Y. *et al.* A Flexible Piezoelectric Strain Sensor Array With Laser-Patterned Serpentine Interconnects. *IEEE Sensors Journal* **20**, 8463–8468 (2020).

S18. Xu, Z. & Lin, Y.-S. A Stretchable Terahertz Parabolic-Shaped Metamaterial. *Advanced Optical Materials* **7**, 1900379 (2019).

S19. Li, J. *et al.* Flexible terahertz metamaterials for dual-axis strain sensing. *Optics Letters,* **38**, 2104–2106 (2013).

S20. Khatib, O., Tyler, T., Padilla, W. J., Jokerst, N. M. & Everitt, H. O. Mapping active strain using terahertz metamaterial laminates. *APL Photonics* **6**, 116105 (2021).

S21. Gutruf, P. *et al.* Mechanically Tunable Dielectric Resonator Metasurfaces at Visible Frequencies. *ACS Nano* **10**, 133–141 (2016).

S22. Filonov, D. S. *et al.* Experimental verification of the concept of all-dielectric nanoantennas. *Applied Physics Letters* **100**, 201113 (2012).

S23. Wheeler, M. S., Aitchison, J. S. & Mojahedi, M. Coupled magnetic dipole resonances in sub-wavelength dielectric particle clusters. *Journal of the Optical Society of America B* **27**, 1083–1091 (2010).

S24. Powell, D. A., Lapine, M., Gorkunov, M. V., Shadrivov, I. V. & Kivshar, Y. S. Metamaterial tuning by manipulation of near-field interaction. *Physical Review B* **82**, 155128 (2010).

S25. Atthi, N. *et al.* Superhydrophobic and superoleophobic properties enhancement on PDMS micro-structure using simple flame treatment method. *Microelectronic Engineering* **230**, 111362 (2020).

S26. Li, G. *et al.* Fabrication of biomimetic superhydrophobic surfaces by a simple flame treatment method. *Polymers for Advanced Technologies* **27**, 1438–1445 (2016).
