# Supplementary material for: A terahertz meta-sensor array for 2D strain mapping
Source: Nat Commun. 2024 Apr 11;15:3157. doi: 10.1038/s41467-024-47474-3 (PMC11009334; doi:10.1038/s41467-024-47474-3)
Supplement: Supplementary file 3 — Description of Additional Supplementary Files [file 41467_2024_47474_MOESM3_ESM.docx]

**Description of Additional Supplementary Files**

**Supplementary Video S1:** Sliding angle demonstration for superhydrophobic sample.

**Supplementary Video S2:** Droplet dynamic impact behaviour shown by the selected droplet vertically impacting the horizontal surface of the sample.

**Supplementary Video S3:** Demonstration of the self-cleaning effect of a superhydrophobic sample with dust as a contaminant.

**Supplementary Video S4:** Demonstration of the hydrophobic properties of the sample surface in the stretched state.
