# Supplementary figures and images for: A terahertz meta-sensor array for 2D strain mapping
Source: Nat Commun. 2024 Apr 11;15:3157. doi: 10.1038/s41467-024-47474-3 (PMC11009334; doi:10.1038/s41467-024-47474-3)

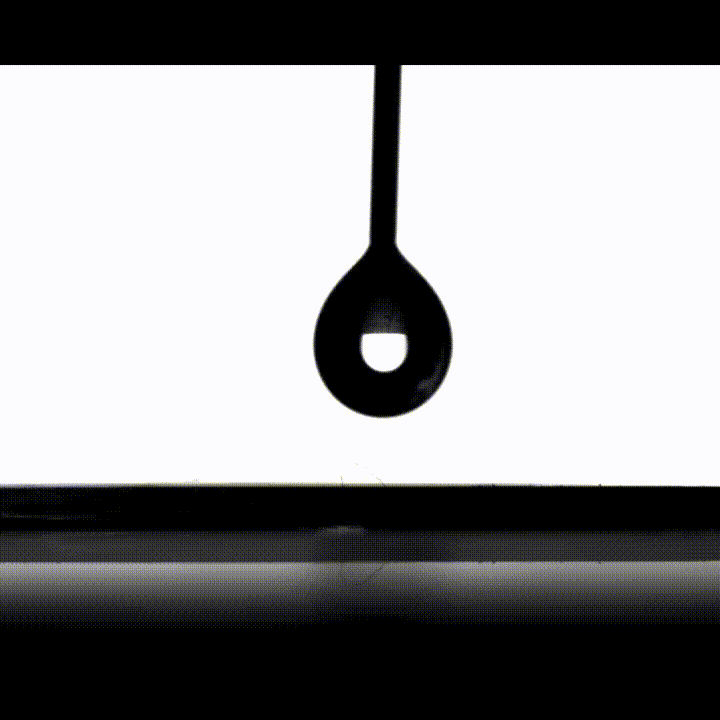

Supplement: Supplementary file 5 — Supplementary Video 2 [file 41467_2024_47474_MOESM5_ESM.gif]
